# Evaluation of the Effect on Sexual Performance of a Nutraceutical Combination Containing Alpha Lipoic Acid, *Vitis vinifera* L. and *Ginkgo biloba*, Compared to Placebo, Avanafil or a Combination of Nutraceutical Plus Avanafil in Males With Type 2 Diabetes Mellitus With Erectile Dysfunction

**DOI:** 10.3389/fendo.2022.847240

**Published:** 2022-04-07

**Authors:** Giuseppe Derosa, Angela D’Angelo, Paola Stefania Preti, Pamela Maffioli

**Affiliations:** ^1^ Centre of Diabetes and Metabolic Diseases, Department of Internal Medicine and Therapeutics, University of Pavia, Pavia, Italy; ^2^ Laboratory of Molecular Medicine, University of Pavia, Pavia, Italy; ^3^ Department of Internal Medicine and Therapeutics, University of Pavia, Pavia, Italy

**Keywords:** alpha lipoic acid, erectile dysfunction, *Vitis vinifera* L, erectile dysfunction (ED), *Vitis vinifera* L., avanafil, metalloproteinases

## Abstract

**Aim:**

To evaluate if therapy with a nutraceutical combination of alpha lipoic acid, Vitis vinifera L. and Ginkgo biloba (Blunorm forte^®^) can be helpful and be synergic with Avanafil.

**Methods:**

The trial included 123 males with type 2 diabetic mellitus and with erectile dysfunction (ED), aged ≥18 years. Patients were divided in four different arms: 1^st^ arm: placebo during the three months of treatment and before sexual act; 2^nd^ arm: placebo for three months and Avanafil: 1 tablet, 200 mg, 15-30 minutes before sexual act; 3^rd^ arm: Blunorm forte: 1 tablet, 40 minutes before the meal (breakfast) during the three months and Avanafil: 1 tablet, 200 mg, 15-30 minutes before sexual act; 4^th^ arm: Blunorm forte: 1 tablet, 40 minutes before the meal (breakfast and dinner) during the three months and placebo 15-30 minutes before sexual act.

**Results:**

A significant reduction of fasting plasma glucose, and homeostasis model assessment-insulin resistance index were recorded both in Avanafil + Blunorm forte and with Blunorm forte. Metalloproteinases-2, and -9 were reduced in the Avanafil + Blunorm forte group. High sensitivity-C-reactive protein was decreased by both Avanafil, and Avanafil + Blunorm forte group. No variations were recorded with the other treatments.

The group treated with Blunorm forte and Avanafil reached a higher International Index of Erectile Function (IIEF) score after 3 months of therapy compared to baseline and placebo and compared to Avanafil and Blunorm forte taken alone.

**Conclusion:**

Blunorm forte^®^ can be helpful and synergic with Avanafil in increasing sexual performance compared to placebo.

## Introduction

Erectile dysfunction (ED) is defined as the inability to attain and maintain penile erection long enough to obtain a satisfactory sexual performance; even if this condition is not lethal, it significantly impacts patients’ quality of life and their psychological well-being (1). Erectile dysfunction has an estimated prevalence between 30% and 50% in general population (2–4), and it is constantly increasing, with a growth forecast of over 300 million of men worldwide suffering from ED by 2025 (5). Erectile disfunction is more prevalent among type 2 diabetic males, obese men, and patients with metabolic syndrome (6). Erectile dysfunction has an earlier onset in type 2 diabetic males, with the diagnosis established 10-15 years earlier compared to not diabetic males. Furthermore, type 2 diabetic males with ED are less responsive to oral pharmacological therapy compared to males with ED without diabetes (7). Several factors seem to play a role in ED development, including vascular, hormonal, neurologic, and psychological factors; lifestyle and aging also have a role (8). Hyperglycemia could cause endothelial damage, starting the pathophysiologic cascade responsible for endothelial dysfunction: endothelial damage impairs vascular nitric oxide synthesis, vasodilatation, and increases inflammation and oxidative stress (9, 10). Data in literature suggest a possible link between lower plasma total testosterone levels and hyperinsulinemia, reduced glucose tolerance, and increased cardiovascular risk both in healthy (11), obese male (12) and males with type 2 diabetes mellitus (13).

We have already published two reports on ED in males with type 2 diabetes mellitus (14, 15), reporting that males with type 2 diabetes mellitus with ED had increased levels of fasting plasma dihydrotestosterone (14).

Alpha lipoic acid seems to have a tangible beneficial effect on ED and on metabolic disorders in males with type 2 diabetes mellitus and can be used to treat such patients. There is evidence that alpha lipoic acid has a certain ability to inhibit the glycosylation of free radicals and can improve vascular endothelial function through endothelial nitric oxide synthase (eNOS) recoupling and increasing the bioavailability of nitric oxide (16). In this way, alpha lipoic acid can play a complementary role with phosphodiesterase type 5 (PDE-5) inhibitors, contributing to nitric oxide production, which is often impossible for diabetic neuropathy patients with severe neurological impairment (16).

Vitis vinifera L. seems to be an inhibitor of PDE-5 enzymes (17). Furthermore, administration of Ginkgo biloba extract in rat model underwent bilateral cavernous nerve injury, increased neuron survival and preserved the neural nitric oxide synthase nerve fibre and contents of the corpus cavernosum. These data suggest the use of Ginkgo biloba extract after radical prostatectomy for repairing the cavernous nerve and recovering of erectile function (18).

In literature, data about the use of nutraceuticals for ED are promising, although very preliminary (19): although medicinal plants serve as a potential source of lead compounds for erectile ED drugs, further studies are warranted to further evaluate their efficacy and safety (20).

All data considered, the aim of this trial was to assess if therapy with a nutraceutical combination of alpha lipoic acid, Vitis vinifera L. and Ginkgo biloba (Blunorm forte^®^) can be helpful and be synergic with Avanafil. Avanafil is a reversible, powerful and highly selective inhibitor of PDE-5 specific for cyclic guanosine monophosphate (cGMP). In occasion of sexual stimulation, there is a local release of nitric oxide, the inhibition of PDE-5 by Avanafil results in an increased cGMP levels in the corpora cavernosa of the penis. This results in a consequent relaxation of the smooth muscles and an increase of blood flow into the genital organ, increasing sexual performance compared to placebo (21). As secondary objective, some hemodynamic and metabolic parameters were evaluated.

## Research Design And Methods

### Study Design

The trial was conducted at the Centre of Diabetes and Metabolic Diseases, Department of Internal Medicine and Therapeutics, University of Pavia and Fondazione IRCCS Policlinico San Matteo, PAVIA, Italy.

Fondazione IRCCS Policlinico San Matteo ethic committee approved the study (P-20160020534). The trial was conducted in accordance with the 1994 Declaration of Helsinki, and its amendments and the Code of Good Clinical Practice. All patients were enrolled after written informed consent to participate was obtained after a full explanation of the study procedures.

### Patients

One hundred and twenty-three males were enrolled.

To be included in the trial, patients needed to respect the following inclusion criteria:

- Being males;
- Being affected by ED, assessed by International Index of Erectile Function (IIEF) questionnaire
- Being affected by type 2 diabetes mellitus according to the ESC (European Society of Cardiology) and EASD (European Association for the Study of Diabetes) Guidelines criteria (22);
- Being aged ≥18 years old.

Suitable patients were identified from review of case notes and/or computerized clinic registers and were contacted by the investigators in person or by phone.

Patients were excluded if they have type 1 diabetes mellitus, LADA or if they had anatomic abnormalities of the penis (Peyronie’s disease) or had undergone gonadectomy. Furthermore, we also excluded hypogonadic subjects and patients treated with drugs for ED such as PDE-5 inhibitors.

The list of medications taken by patients is reported in **Table 1**.

**Table 1:** Drugs taken during the study in the four groups.

Drugn	Placebo (30 subjects)	Avanafil (31 subjects)	Blunorm forte + Avanafil (32 subjects)	Blunorm forte (30 subjects)
***Anti-hypertensives n (%)***				
Diuretic	4 (13.3)	3 (9.7)	4 (12.5)	2 (6.7)
ACE-I	16 (53.3)	13 (41.9)	13 (40.6)	11 (36.7)
Sartan	10 (33.3)	12 (38.7)	14 (43.8)	12 (40.0)
Ca-antagonist	9 (30.0)	10 (32.6)	7 (21.9)	9 (30.0)
β-blocker	3 (6.7)	2 (6.5)	3 (9.4)	1 (3.3)
***Hypo-cholesterolemics n (%)***				
Statin	18 (60.0)	20 (64.5)	17 (53.1)	21 (70.0)
Fibrate	4 (13.3)	5 (16.1)	5 (15.6)	7 (23.3)
Ezetimibe	10 (33.3)	5 (16.1)	11 (34.4)	8 (26.7)
Omega-3	6 (20.0)	4 (12.9)	5 (15.6)	4 (13.3)
PCSK9-I	5 (16.7)	3 (9.7)	3 (9.4)	4 (13.3)
***Anti-aggregants n (%)***				
ASA	5 (16.7)	4 (12.9)	7 (21.9)	6 (20.0)
Clopidogrel	1 (3.3)	2 (6.5)	0 (0.0)	3 (6.7)
***PPI n (%)***	6 (20.0)	6 (19.4)	7 (21.9)	9 (30.0)
***Anti-diabetics n (%)***				
Sulphonylurea	3 (6.7)	2 (6.5)	4 (12.5)	4 (13.3)
Metformin	22 (73.3)	20 (64.5)	24 (75.0)	21 (70.0)
GLP1- RA	5 (16.7)	6 (19.4)	5 (15.6)	4 (13.3)
DPP4-I	7 (23.3)	9 (29.0)	11 (34.4)	10 (33.3)
SGLT2-I	4 (13.3)	5 (16.1)	3 (9.4)	5 (16.7)
Insulin	2 (6.7)	1 (3.2)	0 (0.0)	1 (3.3)

n: number of patients; %: percentage of patients.

ACE-I, Angiotensin Converting Enzyme-Inhibitors; PCSK9-I, Proprotein Convertase Subtilisin/Kexin type 9-Inhibitors; PPI,

Proton Pump Inhibitors; GLP1-RA, Glucagon-Like Peptide 1- Receptor Agonists; DPP4-I, Dipeptidyl Peptidase 4-Inhibitors.

### Treatments

**Figure 1:**
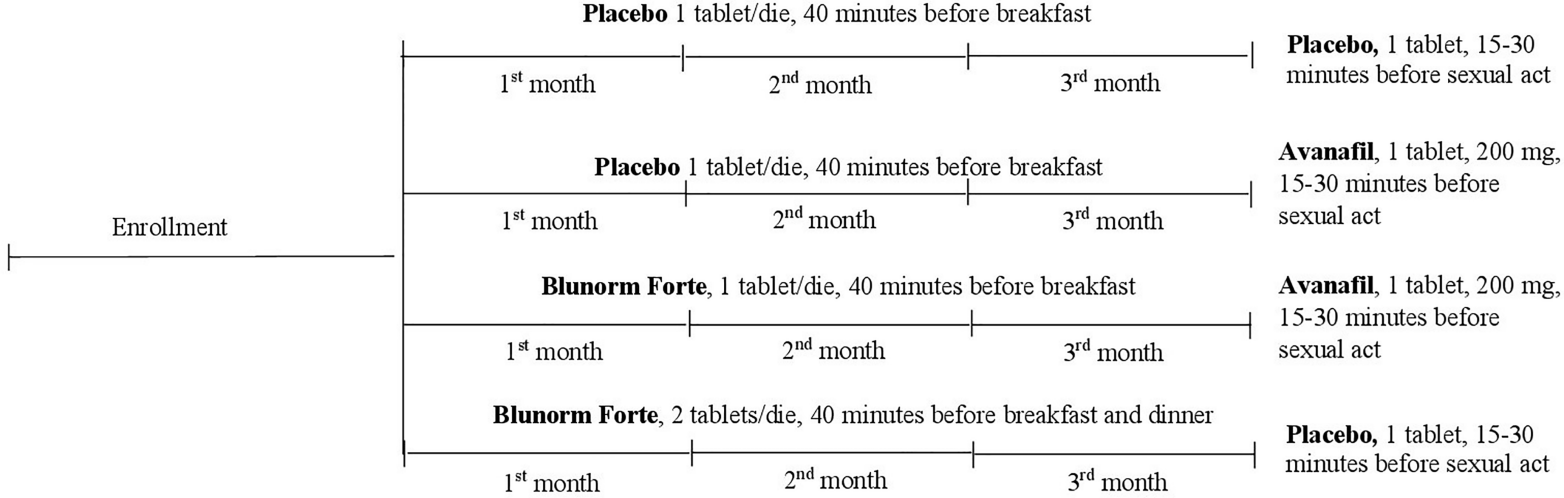
Study Design.

Patients were divided in four different arms (three active and one placebo), in 1:1:1:1 ratio, as followed (**Figure 1**):

1^st^ arm: placebo during the three months of treatment and before sexual act;
2^nd^ arm: placebo for three months and Avanafil: 1 tablet, 200 mg, 15-30 minutes before sexual act;
3^rd^ arm: Blunorm forte: 1 tablet, 40 minutes before the meal (breakfast) during the three months and Avanafil: 1 tablet, 200 mg, 15-30 minutes before sexual act;
4^th^ arm: Blunorm forte: 1 tablet, 40 minutes before the meal (breakfast and dinner) during the three months and placebo 15-30 minutes before sexual act.

Nutraceutical combination composition has been reported in **Table 2**.

**Table 2:** Nutraceutical composition.

Components	Daily intake/tablet	% VNR/tablet*
**Alfa lipoic acid**	400 mg	**-**
**Leucoselect^®^ Fitosoma^®^ * (Vitis vinifera extract)***	200 mg	**-**
**Ginkgoselect^®^ Plus Fitosoma^®^ *(Ginkgo biloba extract)***	80 mg	**-**
**Vitamin B_6_**	1 mg	71
**Vitamin B_12_**	0.5 mcg	20
**Folic acid**	100 mcg	50
**Selenium**	18 mcg	33

*Daily reference nutrient values for vitamins and mineral salts in adults according to Reg. (EU) No 1169/2011.

In this 3-months, double-blind, randomized, clinical trial, patients were instructed to take their first dose of medication on the day after they were given the study medication. All unused medication was retrieved for inventory. All medications were provided free of charge.

### Assessments

The screening assessment included medical history, physical examination, vital signs, and a 12-lead electrocardiogram. We administered patients the IIEF questionnaire to assess erectile function, organ function, sexual desire, and satisfaction level during and after the sexual intercourse. Investigators evaluated: body weight and body mass index (BMI), waist, abdominal, and hip circumferences (WC, AC, and HC, respectively), fasting plasma glucose (FPG), glycated hemoglobin (HbA_1c_), fasting plasma insulin (FPI), homeostasis model assessment insulin resistance index (HOMA-IR), total cholesterol (TC), low density lipoprotein-cholesterol (LDL-C), high density lipoprotein-cholesterol (HDL-C), triglycerides (Tg), systolic blood pressure (SBP), diastolic blood pressure (DBP), testosterone, free testosterone, dihydrotestosterone, sex hormone binding globulin (SHBG), metalloproteinase-2 (MMP-2), metalloproteinase-9 (MMP-9), high sensitivity-C-reactive protein (Hs-CRP), and nitrites/nitrates ratio.

All plasmatic parameters were determined after a 12-h overnight fast. All measurements were performed in a central laboratory. For a description of how various parameters were assessed, see our previous papers (14, 15).

### IIEF Questionnaire

The IIEF questionnaire is composed by 15 items, organized in 5 domains: erectile function, orgasmic function, sexual desire, intercourse satisfaction, and overall satisfaction. IIEF was developed by an international panel of experts, and it has been validated for detecting real treatment effects in patients with ED (23). In the IIEF questionnaire the score for each item ranges from five for normal erection to one for no erection. A score between 26 and 30 means absence of ED, a score between 22 and 25 means mild ED, a score between 17 and 21 means mild to moderate ED, a score between 11 and 16 means moderate ED (11-16), and a score between 6 and 10 means severe ED (24).

### Safety Measurements

Physical examination, vital sign assessment, electrocardiogram, and laboratory tests were performed to safety monitoring. All adverse events were recorded.

### Statistical Analysis

An intent-to-treat analysis was conducted in patients who had received at least one dose of study medication; to be included in the safety analysis, instead, patients needed to have received at least one dose of trial medication after randomization. The null hypothesis was verified using repeated measures analysis of variance (ANOVA) and analysis of covariance (ANCOVA) models (25). The statistical significance of the independent effects of treatments on the other parameters was determined by ANCOVA.

We evaluated sample size using the sample size calculator made available by Epicentro, Istituto Superiore di Sanità (Italy). A difference of at least the 10% compared to the baseline was considered clinically significant; considering an alpha error of 0.05, a confidence level of 95%, and a confidence interval of 8.95, the actual sample size is adequate to obtain a power higher than 0.80 for all variables. Data were presented as mean ± standard deviation.

Statistical analysis of data was performed by means of the SPSS statistical software package for Window (version 14.0; Chicago, Illinois, USA); data are presented as mean ± SD. For all statistical analysis, *P* < 0.05 was considered statistically significant.

## Results

### Study Sample and ED Prevalence

We enrolled 123 males affected by type 2 diabetes mellitus and with ED. The mean age was 61.3 ± 7.4 years in the placebo group, 60.1 ± 7.0 years in the Avanafil group, 59.1 ± 6.4 years in the Blunorm forte + Avanafil group and 60.8 ± 7.2 years in Blunorm forte group. Subjects in the four groups did not differ for age or diabetes duration (**Table 3**).

**Table 3:** Baseline and 3 month results in the four groups of patients.

Parameters	Placebo		Avanafil		Blunorm forte + Avanafil		Blunorm forte	
	Baseline	3 months	Baseline	3 months	Baseline	3 months	Baseline	3 months
N of pts (123)	30	30	31	30	32	30	30	29
Age (years)	61.3 ± 7.4	–	60.1 ± 7.0	–	59.1 ± 6.4	–	60.8 ± 7.2	–
Smoking status	8	8	11	10	14	13	10	10
Diabetes duration (years)	8.2 ± 4.1	–	8.1 ± 4.0	–	8.4 ± 4.3	–	8.2. ± 4.1	–
Hypercholesterolemia duration (years)	7.7 ± 3.8	–	7.4 ± 3.5	–	7.3 ± 3.4	–	7.4 ± 3.5	–
Hypertension duration (years)	8.1 ± 6.1	–	8.3 ± 6.3	–	8.4 ± 6.4	–	8.3 ± 6.1	–
Weight (Kg)	79.2 ± 7.5	79.5 ± 7.7	79.6 ± 7.8	79.2 ± 7.5	78.9 ± 7.3	78.5 ± 7.3	78.8 ± 7.4	78.2 ± 7.0
Height (m)	1.68 ± 0.09	–	1.67 ± 0.08	–	1.68 ± 0.09	–	1.66 ± 0.07	–
BMI (Kg/m^2^)	28.1 ± 2.0	28.2 ± 2.1	28.6 ± 2.4	28.5 ± 2.3	28.0 ± 2.0	27.8 ± 1.9	28.6 ± 2.4	28.4 ± 2.3
WC (cm)	89.5 ± 2.6	89.3 ± 2.4	89.2 ± 2.7	89.1 ± 2.6	88.8 ± 2.1	88.6 ± 1.9	88.5 ± 1.8	88.3 ± 1.7
HC (cm)	101.5 ± 4.5	101.8 ± 4.6	101.6 ± 4.6	101.2 ± 4.3	100.8 ± 4.2	100.6 ± 4.0	100.5 ± 4.0	100.3 ± 4.1
AC (cm)	93.7 ± 3.5	93.4 ± 3.3	93.9 ± 3.3	93.5 ± 3.4	93.1 ± 2.8	93.0 ± 2.7	93.3 ± 3.3	93.2 ± 3.1
SBP (mmHg)	139.8 ± 5.4	139.3 ± 5.7	139.2 ± 5.6	139.3 ± 5.5	139.7 ± 5.2	139.5 ± 5.1	139.4 ± 5.0	139.1 ± 4.9
DBP (mmHg)	83.1 ± 3.9	82.8 ± 3.6	83.3 ± 4.0	83.9 ± 3.7	84.0 ± 3.8	82.1 ± 3.0	82.9 ± 3.7	82.6 ± 3.4
HR (bpm)	75.5 ± 6.3	75.2 ± 6.2	76.2 ± 6.9	76.4 ± 7.0	75.7 ± 6.5	74.7 ± 59	76.1 ± 6.8	75.5 ± 6.3
HbA_1c_ (%)	7.5 ± 1.1	7.3 ± 0.9	7.4 ± 1.0	7.4 ± 1.0	7.5 ± 1.1	7.3 ± 0.9	7.2 ± 0.8	7.3 ± 0.9
FPG (mg/dl)	139.8 ± 18.7	141.1 ± 19.4	140.4 ± 18.9	139.2 ± 18.5	138.7 ± 18.1	130.5 ± 15.5*^	139.9 ± 18.9	131.9 ± 16.9*^
FPI (µU/ml)	15.4 ± 3.9	15.2 ± 3.7	15.5 ± 4.0	15.3 ± 3.8	15.3 ± 3.8	15.5 ± 4.0	15.2 ± 3.7	15.3 ± 3.8
HOMA-IR	5.3 ± 1.2	5.3 ± 1.2	5.4 ± 1.3	5.3 ± 1.2	5.3 ± 1.2	5.0 ± 1.0*^	5.3 ± 1.2	5.0 ± 1.0*^
TC (mg/dl)	188.1 ± 35.2	175.4 ± 33.1	186.4 ± 34.8	173.1 ± 32.8	187.6 ± 34.9	175.8 ± 33.7	187.3 ± 34.6	176.3 ± 33.9
LDL-C (mg/dl)	119.2 ± 20.9	108.4 ± 15.5	117.4 ± 19.6	106.0 ± 14.3	118.4 ± 19.3	106.0 ± 14.7	119.6 ± 21.3	109.0 ± 15.8
HDL-C (mg/dl)	41.3 ± 6.1	41.4 ± 6.2	40.9 ± 5.7	40.6 ± 5.5	41.7 ± 6.4	41.3 ± 6.1	40.6 ± 5.4	40.7 ± 5.6
Tg (mg/dl)	139.5 ± 23.4	128.4 ± 21.7	143.7 ± 22.7	135.3 ± 22.1	138.9 ± 23.1	140.1 ± 23.6	137.5 ± 22.4	135.4 ± 22.2
MMP-2 (ng/ml)	1267.2 ± 131.5	1262.5 ± 127.7	1262.7 ± 127.3	1257.5 ± 124.8	1261.6 ± 125.9	1176.8 ± 111.6*^	1264.7 ± 128.9	1239.1 ± 117.6
MMP-9 (ng/ml)	492.3 ± 56.5	489.7 ± 54.2	489.5 ± 54.0	484.9 ± 52.1	490.7 ± 55.9	462.9 ± 40.1*^	488.3 ± 53.2	483.8 ± 51.7
Testosterone (ng/ml)	7.2 ± 3.8	7.0 ± 3.6	7.4 ± 4.0	7.2 ± 3.6	7.1 ± 3.5	7.3 ± 3.7	7.1 ± 3.5	7.4 ± 3.9
Free Testosterone (pg/ml)	11.6 ± 4.5	11.1 ± 4.2	12.3 ± 4.9	12.5 ± 5.1	11.4 ± 5.3	12.7 ± 5.9	12.1 ± 5.0	12.7 ± 5.5
Dihydrotestosterone (pg/ml)	1.43 ± 0.53	1.40 ± 0.50	1.48 ± 0.61	1.47 ± 0.59	1.46 ± 0.57	1.48 ± 0.60	1.45 ± 0.58	1.45 ± 0.58
SHBG (nmol/l)	32.8 ± 14.3	30.5 ± 12.9	33.1 ± 15.7	32.5 ± 14.1	31.7 ± 12.8	31.2 ± 12.1	33.2 ± 16.1	33.6 ± 16.7
Hs-CRP (mg/l)	1.3 ± 0.4	1.2 ± 0.3	1.2 ± 0.5	1.1 ± 0.4	1.2 ± 0.5	0.8 ± 0.3*^	1.3 ± 0.6	0.9 ± 0.3*^
Nitrites/nitrates µmol/l)	22.8 ± 6.8	24.2 ± 7.3	22.2 ± 6.4	31.3 ± 10.4*	21.9 ± 6.1	38.4 ± 14.7°^	21.6 ± 6.0	33.5 ± 10.7*^

Data are expressed as mean ± standard deviations.

*p < 0.05 vs Baseline; °p < 0.01 vs Baseline; ^p < 0.05 vs Placebo.

BMI, body mass index; WC, waist circumference; HC, hip circumference; AC, abdominal circumference; SBP, systolic blood pressure; DBP, diastolic blood pressure; HR, heart rate; HbA_1c_, glycated hemoglobin; FPG, fasting plasma glucose; FPI, fasting plasma insulin; HOMA-IR, homeostasis model assessment-insulin resistance; TC, total cholesterol; LDL-C, low density lipoprotein-cholesterol; HDL-C, high density lipoprotein-cholesterol; Tg, triglycerides; MMP-2, metalloproteinases-2; MMP-9, metalloproteinases-9; SHBG, sex hormone binding globulin; Hs-CRP, high sensitivity-C-reactive protein.

### Anthropometric Variables

No difference among the four groups were recorded regarding body circumferences, body weight and BMI.

### Glyco-Metabolic Control

There was a significant decrease of FPG, and HOMA-IR both in Avanafil + Blunorm forte and with Blunorm forte compared to baseline and placebo (p < 0.05 for both). No differences were recorded among groups regarding FPI, and HbA_1c_.

Investigators did not record any differences in lipid profile between the two groups.

### Sexual Hormones

Sexual hormones did not change during the study with neither treatment.

### Cytokines and Metalloproteinases

Metalloproteinases-2, and MMP-9 were reduced in the Avanafil + Blunorm forte group, both compared to baseline, and to placebo (p <  0.05 for both). Hs-CRP was decreased by both Avanafil, and Avanafil + Blunorm forte group. No variations were recorded with the other treatments.

Regarding nitrites/nitrates, there was an increase of nitrites/nitrates with avanafil (p < 0.05), with Avanafil + Blunorm forte (p < 0.01), and with Blunorm forte (p < 0.05) compared to baseline. Nitrites/nitrates were higher with Avanafil + Blunorm forte, and with Blunorm forte compared to placebo (p < 0.05 for both).

### IIEF

**Figure 2:**
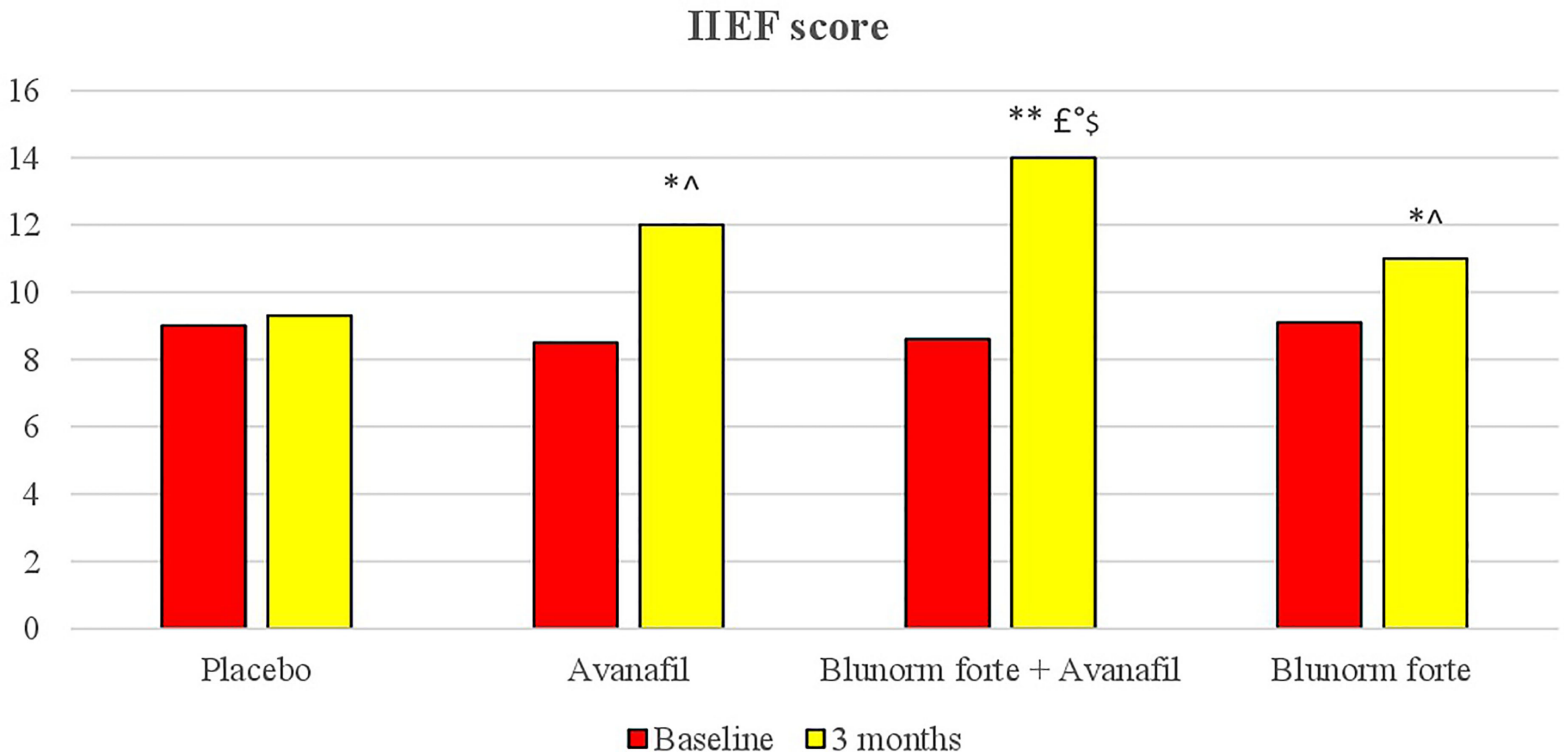
IIEF (International Index of Erectile Function) questionnaire at baseline and after treatments. *p < 0.05 vs baseline; **p < 0.01 vs baseline; ^p < 0.05 vs placebo group; ^£^P < 0.01 vs placebo group; °p < 0.05 vs Avanafil group; $p < 0.05 vs Blunorm forte goup. Score from 22 to 25: normal sexual activity. Score from 17 to 21 mild erectile dysfunction. Score from 12 to 16 mild-moderate erectile dysfunction. Score from 8 to 11 moderate erectile dysfunction. Score from 5 to 7 severe erectile dysfunction.

The group treated with Blunorm forte and Avanafil reached a higher IIEF score after 3 months of therapy both compared to baseline and placebo (p < 0.01 for both), and vs Avanafil and Blunorm forte taken alone (p < 0.05 for both) (**Figure 2**).

## Discussion

Among diabetic complications, peripheral neuropathy is very common, with a prevalence rate of 5.3-47.6% for peripheral sensorimotor neuropathy (26). Peripheral neuropathies include not only sensory loss and motor deficits, but also autonomic neuropathy, such as ED. Oxidative stress plays a main role in the pathogenesis of diabetic neuropathy (27). Alpha lipoic acid improves nerve blood flow, reduces oxidative stress and improves distal nerve conduction in a rat model of diabetic neuropathy (28), suggesting promising results in terms of treatment of neuropathic symptoms (29, 30). The study conducted by Agathos et al. showed that 600 mg/day alpha-lipoic acid significantly impact the control of neuropathic symptoms, improving quality of life (31). Reactive oxygen species (ROS) and reactive nitrogen species (RNS) contribute to oxidative stress. Diabetes impairs eNOS activity, increasing ROS production, with a consequent reduction of nitric oxide bioavailability. Regarding the effects of alpha lipoic acid on oxidative markers, Derosa et al. already conducted a study aimed to evaluate the effects of alpha lipoic acid on oxidation in patients with type 2 diabetes mellitus (32) showing that alpha lipoic acid seems to improve markers of oxidative stress, such as glutathione peroxidase (GSH-Px), superoxide dismutase (SOD), and malondialdehyde (MDA), and of inflammation (Hs-CRP). According to this previous trial, we also showed that the nutraceutical containing alpha lipoic acid decreases MMP-2, and MMP-9 and increases nitrites/nitrates ratio. Nitrites/nitrates ratio is inversely proportional to oxidative cell damages and granulocytic apoptosis (33); reducing oxidative stress can help in reducing endothelial damage with a role in ED. Regarding the effects on sexual performance, when the nutraceutical was added to Avanafil, it improved IIEF score, when compared to Avanafil alone. This action is probably due to Vitis vinifera L., which seems to be an inhibitor of PDE-5 enzymes (17). These data suggest that the assumption of Blunorm forte^®^ can be helpful in patients with ED in reducing chronic endothelial damage and oxidative stress, improving Avanafil action on erection.

Regarding study limitations, anti-hypertensive drugs could have a negative effect on ED, patients were taking different medications, but, given the small sample size, we could not stratified the results according to the drugs taken; however, anti-hypertensive drugs were not modified during the study. Another limitation is that we did not consider hyperhomocysteinemia among the possible risk factors for ED, despite the fact that the nutraceutical contains folic acid. Finally, a possible presence of small traces of PDE-5 inhibitor has been reported in some herbal products (34). We did not perform chromatography and spectroscopy of the nutraceutical combination used, so this can not be excluded.

## Conclusions

We can conclude that a nutraceutical combination of alpha lipoic acid, Vitis vinifera L. and Ginkgo biloba (Blunorm forte^®^) can be helpful and synergic with Avanafil in increasing sexual performance compared to placebo. This study certainly shows that Avanafil administered before sexual intercourse is significantly effective in erection compared to Blunorm forte and clearly compared to placebo, but in combination with Blunorm forte there is also a significant improvement compared to the group with Avanafil alone. This could be translated into a possible chronic intake of Blunorm forte and that, therefore, males with type 2 diabetes mellitus could start and continue the treatment for a further improvement of endothelial function, as demonstrated by the improvement of FPG, Hs-CRP and nitrites/nitrates ratio in the Blunorm forte group and take Avanafil at the time of sexual intercourse for further enhancement. Blunorm forte was well tolerated at all stages of the study and did not interfere with other medications the patients were taking.

## Data Availability Statement

The original contributions presented in the study are included in the article/supplementary material. Further inquiries can be directed to the corresponding author.

## Ethics Statement

The studies involving human participants were reviewed and approved by Fondazione IRCCS Policlinico San Matteo. The patients/participants provided their written informed consent to participate in this study.

## Author Contributions

Design and conduction of the study: GD. Data collection: all authors. Data interpretation and manuscript writing: all Authors. All authors read and approved the final version of the manuscript.

## Funding

The authors did not receive any funding for carrying out the clinical study. The investigational products were provided by the manufacturer Uriach S.p.A. (Italy) free of charge.

## Conflict of Interest

The authors declare that the research was conducted in the absence of any commercial or financial relationships that could be construed as a potential conflict of interest.

## Publisher’s Note

All claims expressed in this article are solely those of the authors and do not necessarily represent those of their affiliated organizations, or those of the publisher, the editors and the reviewers. Any product that may be evaluated in this article, or claim that may be made by its manufacturer, is not guaranteed or endorsed by the publisher.
